# Machine Learning-Assisted Optimisation of the Laser Beam Powder Bed Fusion (PBF-LB) Process Parameters of H13 Tool Steel Fabricated on a Preheated to 350 ^∘^C Building Platform

**DOI:** 10.3390/ma19010210

**Published:** 2026-01-05

**Authors:** Katsiaryna Kosarava, Paweł Widomski, Michał Ziętala, Daniel Dobras, Marek Muzyk, Bartłomiej Adam Wysocki

**Affiliations:** 1 Faculty of Mathematics and Natural Sciences, Cardinal Stefan Wyszynski University in Warsaw, 01-938 Warszawa, Poland; k.kosarava@uksw.edu.pl; 2 Center for Materials Engineering and Metal Forming, Wrocław University of Science and Technology, 50-371 Wrocław, Poland; 3 Lukasiewicz—Warsaw Institute of Technology, 01-796 Warszawa, Poland; 4 Department of Mechanical Engineering, Wrocław University of Science and Technology, 50-371 Wrocław, Poland; 5 Multidisciplinary Research Centre, Cardinal Stefan Wyszynski University in Warsaw, 05-092 Dziekanów Leśny, Poland

**Keywords:** additive manufacturing, machine learning, regression, powder bed fusion using laser beam (PBF-LB), porosity prediction, parameters optimisation, H13 tool steel, building platform preheating

## Abstract

This study presents the first application of Machine Learning (ML) models to optimise Powder Bed Fusion using Laser Beam (PBF-LB) process parameters for H13 steel fabricated on a 350 °C preheated building platform. A total of 189 cylindrical specimens were produced for training and testing machine learning (ML) models using variable process parameters: laser power (250–350 W), scanning speed (1050–1300 mm/s), and hatch spacing (65–90 μm). Eight ML models were investigated: 1. Support Vector Regression (SVR), 2. Kernel Ridge Regression (KRR), 3. Stochastic Gradient Descent Regressor, 4. Random Forest Regressor (RFR), 5. Extreme Gradient Boosting (XGBoost), 6. Extreme Gradient Boosting with limited depth (XGBoost LD), 7. Extra Trees Regressor (ETR) and 8. Light Gradient Boosting Machine (LightGBM). All models were trained using the Fast Library for Automated Machine Learning & Tuning (FLAML) framework to predict the relative density of the fabricated samples. Among these, the XGBoost model achieved the highest predictive accuracy, with a coefficient of determination $R^{2}=0.977$, mean absolute percentage error MAPE = 0.002, and mean absolute error MAE = 0.017. Experimental validation was conducted on 27 newly fabricated samples using ML predicted process parameters. Relative densities exceeding 99.6% of the theoretical value (7.76 g/cm^3^) for all models except XGBoost LD and KRR. The lowest MAE = 0.004 and the smallest difference between the ML-predicted and PBF-LB validated density were obtained for samples made with LightGBM-predicted parameters. Those samples exhibited a hardness of 604 ± 13 HV0.5, which increased to approximately 630 HV0.5 after tempering at 550 °C. The LightGBM optimised parameters were further applied to fabricate a part of a forging die incorporating internal through-cooling channels, demonstrating the efficacy of machine learning-guided optimisation in achieving dense, defect-free H13 components suitable for industrial applications.

## 1. Introduction

Steel is the most widely used metallic material in the world, playing a crucial role across sectors such as construction (beams, reinforcement bars), transportation (automotive frames, shipbuilding), infrastructure (bridges, pipelines), and machinery. According to the World Steel Association, over 1.8 billion tonnes of steel are produced annually as of the early 2020s, significantly outpacing the production of aluminium, copper, or titanium. H13 tool steel, a versatile chromium-molybdenum alloy, is commonly utilised in demanding industrial applications, particularly where high temperatures are involved. Its primary uses include hot forging, die casting, extrusion, and the creation of moulds and dies for injection moulding. H13 is valued for its remarkable resistance to heat, wear, and thermal fatigue [1,2]. Traditionally, H13 is manufactured through processes such as melting, casting, forging, and heat treatment, all of which optimise its mechanical properties and stability. It is known for its excellent combination of hardness, toughness, and ability to maintain strength at elevated temperatures, often up to 600 °C. These attributes make H13 a preferred choice in the tooling, aerospace, and automotive industries, where reliability and durability are essential. On the other hand, H13 steel is difficult to weld due to its high content of carbide-forming elements and high hardenability, both of which result from its chemical composition. These two characteristics result in a hard, brittle microstructure during welding, thereby increasing the material’s susceptibility to cracking. Furthermore, the amount of heat introduced during the welding process leads to the softening of the microstructure, thus reducing its mechanical properties [3,4]. Therefore, the microstructure and mechanical properties of H13 steel after various fabrication methods remain the subject of ongoing research.

Nowadays, H13 steel is also produced using various additive manufacturing technologies outlined in ASTM/ISO 52900:2021 [5]. One notable method is Direct Energy Deposition (DED), one of the seven Additive Manufacturing (AM) technologies defined in this standard. DED is effective for fabricating functionally graded materials (FGMs) and for repairing high-value dies. Recent studies indicate that intrinsic heat treatment during DED creates a graded microstructure that improves wear resistance [6]. The Wire Arc Additive Manufacturing (WAAM) process, a type of DED technology, offers deposition rates of 1–4 kg/h. This makes WAAM particularly suitable for manufacturing hot-working tools. Tooling made by WAAM with integrated conformal channels can reduce part mass by about 25% using strategically placed voids without losing structural integrity [7]. However, DED processes face limitations related to channel diameter, which must be several millimetres. Additionally, producing features with highly inclined surfaces is also difficult, and additional machining is often required to achieve the desired surface topography.

The attainment of optimal geometrical freedom and precision in AM is predominantly realised through powder-in-bed technlogies, notably Binder Jetting (BJT) and Powder Bed Fusion (PBF). In the case of BJT, it has been demonstrated that a density of 95% for H13 steel can be achieved, alongside a hardness value of 499 HV10, following the sintering of green parts within a temperature range of 1410–1415 °C [8]. This methodology capitalises on the utilisation of fine powders, characterised by diameters less than 10 μm. In contrast, market availability of such fine metallic powders is somewhat constrained, as most commercially available options possess diameters ranging from 30 to 70 μm. Furthermore, a density below 99% of the theoretical density limits its use in some more demanding applications. Conversely, Powder Bed Fusion using a Laser Beam (PBF-LB) stands out as a process that can generate stainless steel cellular structures with densities exceeding 99.5% [9]. However, the problems addressed in PBF-LB processes still include process parameter optimisation, density and porosity prediction, defect detection, and melt pool geometry prediction.

AI and machine learning approaches are increasingly being utilised to tackle these challenges. Liu et al. [10] conducted a comprehensive review of ML applications in PBF-LB processes, examining the interconnections among process parameters, microstructure, mechanical properties, and overall performance. This review highlighted the substantial potential of various ML techniques for optimising additive manufacturing processes and accurately predicting material properties. Current artificial intelligence (AI) models can recommend optimal material selections and process configurations to achieve the desired product performance, while also facilitating the development of innovative composites and improving mechanical behaviour through a deeper understanding of material physics [11]. In additive manufacturing, traditional machine learning models have been widely used to predict porosity and density. These include Linear Regression (LR), Decision Trees (DT), Support Vector Machines (SVM), and Random Forests Regression (RFR) have been employed to predict the porosity and density of various steels. These models typically utilise hand-engineered features, including laser power “P” [W], scanning speed “V” [mm/s], distance between scanning vectors “H” [μm], and layer thickness “T” [μm], and perform effectively on relatively small, structured datasets [12]. For property prediction, studies have successfully employed classical ML models to predict ultimate tensile strength and elongation [13] and deep neural networks to link in situ photodiode signals with mechanical performance in PBF-LB parts [14]. AI is used to predict the fatigue life of metals and to analyse the factors that influence it, including material properties, manufacturing processes, service conditions, environmental factors, and operational usage [15,16,17]. Process parameter-based ML models have achieved over 99% accuracy in predicting part density across metals such as aluminium and nickel alloys [18]. Benchmarking studies have further strengthened the field by evaluating ML performance across diverse materials and machines [19]. Melt pool geometry, a critical factor in determining microstructure, has also been predicted using ML models [20] and experimental ML pipelines like MeltpoolNet [21]. Deep learning models based on artificial neural network (ANN) architectures can automatically extract complex features from large, unstructured data, such as images, sensor streams, or process videos, capturing intricate patterns, including defect formation and surface quality variations, that classical models might miss. ANN showed high potential for real-time defect detection in the PBF-LB process, aiming to improve product quality and process reliability [22]. They can infer internal flaws from surface features [23] and segment microstructural defects, such as lack of fusion or porosity, in samples [24]. Deep neural networks can analyse thermographic images of samples to detect defects [25], achieving 96.8% accuracy in detecting porosity/cracks. Together, these advances demonstrate AI/ML’s vast potential to enable predictive, adaptive, and intelligent AM systems.

This study focuses on selecting parameters for the PBF-LB process, which is essential for industry, especially when working with new materials or transitioning between feedstock suppliers. Such parameter selection represents the initial stage of optimising the PBF-LB process. Currently, identifying process parameters that effectively minimise porosity (a material property that significantly affects mechanical properties) is both time-consuming and resource-intensive. Typically, this optimisation process involves adjusting multiple parameters and producing a substantial number of samples for characterisation. The primary objective of our research is to assess the effectiveness of various ML models for predicting PBF-LB process parameters and achieving densities that closely match theoretical values, particularly for H13 steel, which poses challenges during welding. We introduce the application of eight ML models to predict the PBF-LB process parameters that yield the lowest porosity in H13 steel, specifically on building platforms preheated to 350 250 °C. In our study, the preheating temperature exceeds the 200–250 °C range, which is typically the maximum for industrial AM machines. We have performed the study on building table preheated to higher temperatures because H13 tool steel has high hardenability and is prone to cracking at high cooling rates (approximately $10^{5}$ K/s), typical of laser-based processing [26]. Furthermore, heating the platform to 350 °C minimises thermal gradients, facilitates hydrogen removal [27], and reduces the potential for crack formation, while ensuring that temperatures do not exceed 450 °C, which could negatively affect martensitic transformation [28]. This approach enhances the stability of the fabrication process and improves microstructural control compared to lower preheating temperatures.

Eight ML models were verified in this study: 1. Support Vector Regression (SVR), 2. Kernel Ridge Regression (KRR), 3. RFR, 4. Stochastic Gradient Descent (SGD), 5. Extreme Gradient Boosting (XGBoost), 6. Extreme Gradient Boosting with limited depth (XGBoost LD), 7. Extra-Trees Regressor (ETR), and 8. Light Gradient Boosting Machine (LightGBM). To identify the best-performing model, we utilised the Fast Library for Automated Machine Learning & Tuning (FLAML) framework, which autonomously optimises hyperparameters for ML models beyond predetermined ranges [https://microsoft.github.io/FLAML/ accessed on 31 December 2025]. A thorough evaluation of the trained models, for porosity prediction, was conducted using $R^{2}$, Mean Absolute Percentage Error (MAPE), and Mean Absolute Error (MAE) metrics. This multi-metric assessment provides a comprehensive view of model performance, as each metric highlights different facets of accuracy. This allows us not only to gauge how well models account for the overall variation in the data but also to evaluate the significance of prediction errors. All eight tested ML models were validated for their predictive capability in the PBF-LB process by fabricating H13 steel samples. Notably, a single set of PBF-LB process parameters predicted by an ML model yielded laboratory samples with Archimedes’ density closest to the theoretical H13 value. This ML model (PBF-LB parameters set) was used to fabricate a more complex component from H13 tool steel: a forging die punch with integrated cooling channels.

## 2. Materials and Methods

### 2.1. Powder Characterisation Methods

The material used for the experiment was H13 tool steel, manufactured by Höganäs Belgium S.A., Ath, Belgium According to the manufacturer’s information, the average particle size ranged from 15 to 53 μm. The chemical composition of the H13 steel, as provided in the manufacturer’s data sheet, is presented in Table A1 of Appendix A.1. To verify the quality of the powder, particle size and shape analysis were conducted using a Kamika mini 3D analyser (Kamika Instruments, Warsaw, Poland). Additionally, the powder flow coefficient was measured using an Anton Paar MCR 302e rheometer (Anton Paar Ltd., Luton, Bedfordshire, UK). The H13 powder included in the resin was subjected to metallographic preparation, which involved grinding on SiC papers with granulations ranging from 400 to 4000, followed by polishing with 1 μm diamond suspension and 0.1 μm silica suspension. Microstructure observations after etching with Nital reagent (3% HNO_3_ + 97% CH_3_CH_2_OH) and the morphology of H13 steel powder without metallography preparation were carried out using an Axia ChemiSEM (Thermo Fisher Scientific, Waltham, MA, USA) Scanning Electron Microscope (SEM).

### 2.2. Design of PBF-LB Experiment

**Figure 1:**
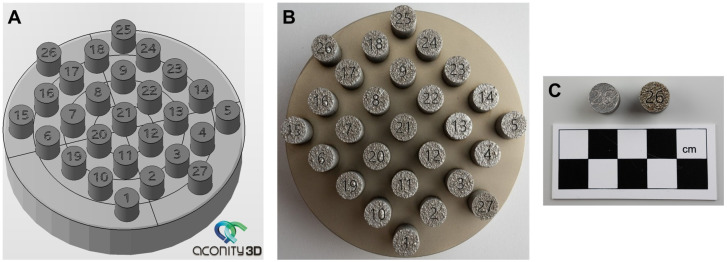
Samples CAD model visualisation in Autodesk Netfabb Premium 2024.0 (**A**), platform P3 fabricated using the PBF-LB process on a building platform preheated 350°C (**B**), Sample 26 from platform 7–100 μm “Mark depth” and platform 5–500 μm “Mark depth” (**C**).

The H13 steel PBF-LB fabrication process was conducted using the Aconity mini device (Aconity3D GmbH, Herzogenrath, Germany) at the Multidisciplinary Research Centre (Dziekanów Leśny, Poland). During the multiple processes, 189 cylindrical samples (10 mm in diameter and 10 mm in height) were fabricated and used as a dataset for training and testing ML models (Platforms 1–7). Several PBF-LB parameters were adjusted during the fabrication of the ML data set. These included laser power (250–350 W), scanning speed (1050–1300 mm/s), laser spot size (65–85 μm), and the distance between scanning vectors (70–90 μm). The layer thickness was maintained at 60 μm, while the platform was heated to 350 °C. This high-temperature building platform temperature was kept during fabrication to minimise stresses [29] and facilitate the melting process of the challenging-to-weld steel grade H13 [30]. Each set of parameters was used to fabricate at least 3 samples, which were randomly placed on the building platform for statistical purposes. On each building platform, the number of samples was always 27, with the samples numbered on the top surface (see Figure 1A,B). We utilised information about the placement of the samples on the building platforms to train ML models. However, we didn’t use this information to select the PBF-LB process settings that yield the minimum porosity in H13 steel. Table 1 presents the range of parameters used along with their steps. The specific parameter ranges for each build plate, along with the sample numbering on the building platforms, are provided in the Supplementary Material spreadsheets (Supplementary Material).

An additional platform (No. 8) was created using 27 samples with PBF-LB process parameters predicted by the eight ML models (Table 6 in Section 3.3, ML results), selecting from each model the set that resulted in the highest theoretical density and the lowest energy density, “E” [J/mm^3^]. This was done to empirically verify the performance of ML models in predicting the density of H13 steel and to select the PBF-LB parameter set for fabricating a more complex element—a part of the forging die with cooling channels.

### 2.3. Samples Characterisation

#### 2.3.1. Archimedes’ Theoretical Density and X-Ray Computed Micro-Tomography (XCT)

Archimedes’ method was used to determine the theoretical density and porosity of all samples using a RADWAG AS 520.X2 PLUS (Radwag, Radom, Poland) balance (d = 0.1 mg). The theoretical density of 7.76 g/cm^3^ was used for H13 steel in the calculations. Additionally, the porosity of the die fabricated using PBF-LB parameters predicted by the ML model, which yielded the density closest to the theoretical value for H13 steel, was verified using an XCT Nikon XT H 225 ST 2x (Nikon Corporation, Tokyo, Japan). The source voltage and source current to obtain XCT scans were set to 215 kV and 830 μA, respectively. A 2.5 mm copper filter material was selected to achieve the optimal greyscale value for the reconstruction. The scanning procedure was performed by acquiring 4000 projections, each with 1 frame, at an exposure time of 125 milliseconds. The reconstructed voxel size was set to 13.4 μm. The reconstruction data were prepared using Nikon CT Pro 3D XT 6.9.1 software. The data visualisation and porosity measurements were performed using Volume Graphics Studio Max 2022.3 with the Additive Manufacturing plugin set (Volume Graphics GmbH, Heidelberg, Germany).

#### 2.3.2. Heat Treatments and Metallographic Preparation

**Figure 2:**
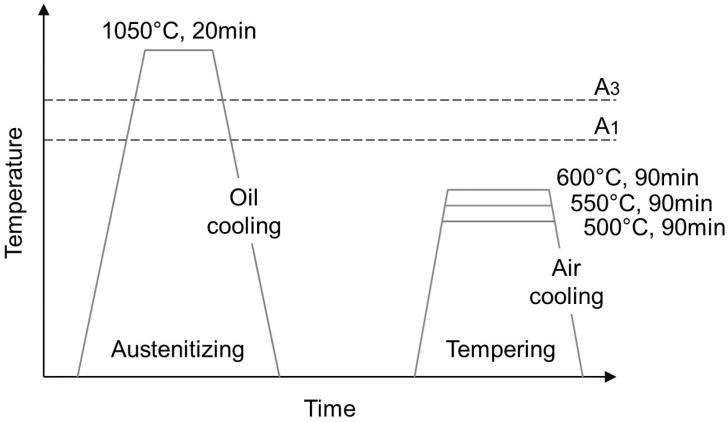
Scheme of the heat treatments performed in the research.

Cylindrical samples for heat treatment were manufactured using parameters predicted by the ML model, yielding a density closest to the theoretical value for H13 steel. These samples underwent heat treatment, which included two approaches: 1. tempering by holding for 90 min at three different temperatures (500 °C, 550°C, and 600°C) and cooling in air; 2. oil quenching followed by heating to the austenite temperature of 1050°C and holding for 20 min + tempering after holding for 90 min at three different temperatures (500 °C, 550 °C, and 600°C) and cooling in air (Table 2 and Figure 2). The purpose of the heat treatments was to provide the necessary post-processing after the PBF-LB process, enabling the target components (forging dies) to achieve the microstructure required for operation at temperatures ranging from 400 to 600 °C. The microstructure of the samples after heat treatment was verified by metallographic examination of cross-sections. To prepare for metallographic observation, the samples were first cut in half parallel to the build direction (perpendicular to the layers).

The inner surfaces of the samples were mechanically ground and polished with a diamond suspension, then etched with Nital reagent (2% HNO_3_+ 98% CH_3_CH_2_OH), which had a slightly lower nitric acid concentration than that used for powder etching. The microstructure was examined using an Olympus GX51 optical microscope (Olympus Corporation, Hachiōji, Tokyo). The Vickers microhardness was measured using the LECO LM100AT (LECO Corporation, St. Joseph, MI, USA) hardness tester at a load of 500 g. Six hardness measurements were made for each sample. The presented hardness result is the average of the measurements. The final fragment of the forging tool, featuring an internal channel was subsequently examined under a light microscope Keyence VHX-7000 (KEYENCE International, Machelen, Belgium).

### 2.4. ML Models

#### 2.4.1. Data Analysis and Preprocessing

The total number of data samples fabricated in the PBF-LB process was 189, which corresponds to 7 platforms with 27 samples each in each bath. From this dataset, 7 samples showing the lowest density values within their respective process parameters were removed. The collected data was split into training and test sets at an 80:20 ratio, with 145 samples in the training set and 37 in the test set. To prevent model overfitting, another operator obtained repeated theoretical Archimedes density measurements for platform no.6 (samples marked as 6.1 in Supplementary Material). These data were split to ensure that paired measurements from the same sample were consistently allocated to the training set or, analogously, to the test set. Finally, the training and testing sets contained 166 and 43 samples, respectively. The Kolmogorov–Smirnov tests [31] demonstrated that both the training and test sets are derived from the same underlying distribution. For all features examined, the tests produced *p*-values exceeding 0.2, suggesting a high likelihood that any observed differences between the training and test samples are attributable to random variation. This provides no justification for rejecting the null hypothesis of identical distributions. Additionally, the analysis of Wasserstein distances reinforces this conclusion, as these distances remain small in relation to the respective feature ranges. Specifically, the Wasserstein distances range from 0.28% to 5.7% of the mean values of the features. Overall, the train/test split was statistically balanced with respect to both the target variable and the selected predictors. To identify the PBF-LB process parameters that maximise theoretical density and minimise porosity using ML models, we followed several steps. First, we analysed the available data to understand its characteristics, structure, and potential issues, including missing values and outliers. The data was then pre-processed to prepare it for training ML models.

**Figure 3:**
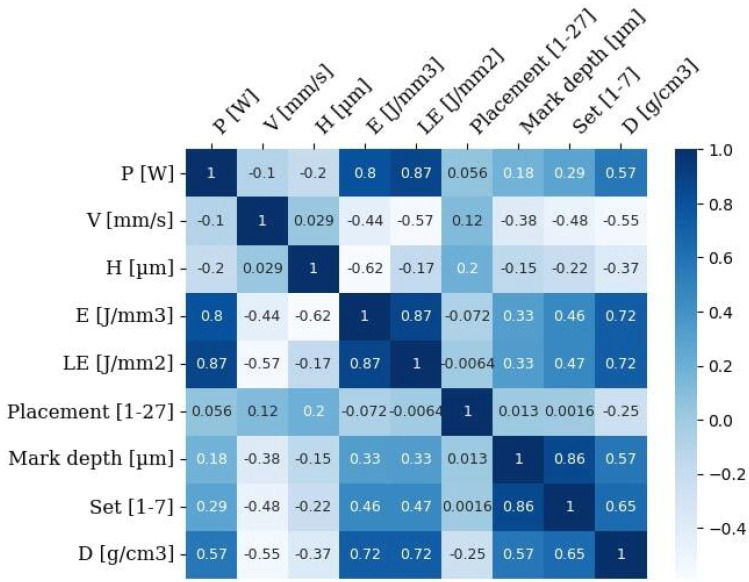
Correlation matrix between PBF-LB parameters and sample density.

The preprocessing stage involved several steps to prepare the data for modelling. First, three features—layer thickness “T” [μm], building platform preheating temperature “Temp.” [°C] and laser spot size “F” [μm] were removed because they were constant and don’t correlate with the target variable, alloy theoretical density “D” [g/cm^3^]. The correlation between process parameters (laser power “P” [W], scanning speed “V” [mm/s], hatch spacing “H” [μm], volumetric energy density “E” [J/mm^3^], linear energy density “LE” [J/mm^2^], sample position on the build platform (marked on each sample by number on the top surface) “Placement”, and sample marking depth “Mark depth”) and the target property, theoretical density “D” [g/cm^3^], was analysed to establish the relative significance of the individual parameters (Figure 3). Correlation analysis revealed significant relationships between theoretical density “D” [g/cm^3^], volumetric energy density “E” [J/mm^3^], and linear energy density “LE” [J/mm^2^], which were calculated using Formula (1). Additionally, correlations were noted with laser power “P” [W], depth of sample marking “Mark depth”, and the platform number “Set”. However, the correlation with the platform number “Set” parameter was found to be insignificant, as the last experiments were conducted using PBF-LB process parameters that yielded the lowest standard deviation in Archimedes measurements results. The relatively low inter-feature correlations suggest that there is no strong redundancy among predictors, allowing all features to be retained for modelling. This indicates that more complex, non-linear approaches might be better suited to capture patterns in the data.

(1) $$\begin{array}{ccc} E[{J/\mathrm{mm}}^{3}] & = & \frac{P[W]}{V\left[\mathrm{mm}/s\right]\times H\left[m\right]\times T\left[m\right]\times 10^{-6}},\\ LE[{J/\mathrm{mm}}^{2}] & = & \frac{P[W]}{V\left[mm/s\right]\times T\left[m\right]\times 10^{-3}}. \end{array}$$

The following preprocessing step is data encoding and normalisation. Categorical features—specifically, the depth of sample marking “Mark depth” (0.5 mm—A or 0.1 mm—B, see Supplementary Material) and the position of each sample on the building platform “Placement” (number on the top of the sample in a range 1–27) were encoded as integers to facilitate numerical processing. Encoding in our study means converting non-numeric data, such as categories, labels, or text, into a numerical format that ML algorithms can understand. Normalisation was then applied to the numeric columns using the MinMaxScaler. Normalisation transforms numerical features to a similar range, preventing features with large values from dominating the model and ensuring balanced contributions and better performance in scale-sensitive ML algorithms.

Subsequently, we trained various machine learning models on this dataset and assessed their performance using the $R^{2}$ coefficient. For all eight models, we predicted the density of H13 tool steel across different combinations of laser parameters. We identified nine optimal laser settings (as outlined in Table 6 of Section 3.3) that resulted in the highest predicted sample density while maintaining the lowest energy density “E“[J/mm^3^] for each model. Based on these predicted parameters, we conducted physical PBF-LB experiments to fabricate the corresponding samples. Finally, we evaluated the density and microstructure of the fabricated samples, ultimately selecting a set of parameters for producing a representative piece of the H13 steel die with conformal channels.

#### 2.4.2. Model Description

In this section, we provide a brief description of each model used in our research, along with its key characteristics.

**I. Kernel methods**:

1. *Support Vector Regression (SVR)*—An extension of SVM that can be used to solve regression problems. It optimises a function by finding a tube that approximates a continuous-valued function while minimising the prediction error [32].
2. *Kernel Ridge Regression (KRR)*—Combines Ridge regression and classification (linear least squares with L2-norm regularisation) with the kernel trick. This approach allows the model to learn a linear function in the feature space induced by the chosen kernel and the data. For non-linear kernels, this results in a non-linear function in the original input space. The form of the model learned by Kernel Ridge Regression is similar to that of SVR. However, they use different loss functions: KRR employs squared error loss, while SVR utilizes an ε-insensitive loss, both combined with L2 regularisation [33].

**II. Linear model with regularisation:**

3. *Stochastic Gradient Descent (SGD) Regressor*—Linear model fitted by minimising a regularised empirical loss with stochastic gradient descent. It implements a plain stochastic gradient descent learning routine, which supports different loss functions and penalties to fit linear regression models.

**III. Tree-based ensemble models:**

**Figure 4:**
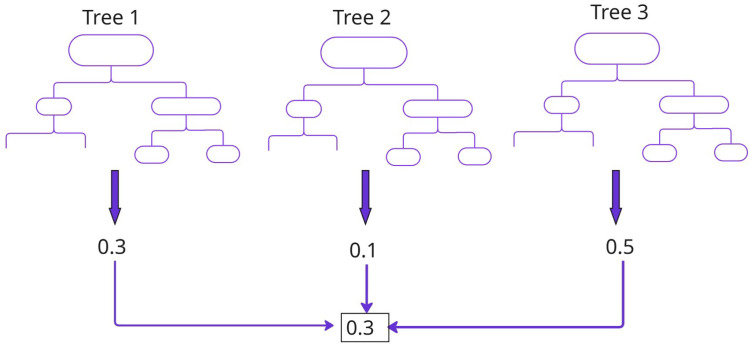
Ensemble model for regression.

4. *Random Forest Regressor (RFR)*—A random forest is an ensemble learning method (Figure 4) that combines the predictions from multiple decision trees to produce a more accurate and stable prediction. RFR predicts continuous values by averaging the results of multiple decision trees.
5. *Extreme Gradient Boosting (XGBoost)*—An optimised distributed gradient boosting library designed to be highly efficient, flexible and portable. XGBoost builds an ensemble of weak decision tree models iteratively, optimising each new tree to correct the errors of the previous ones. It includes L1 (Lasso) and L2 (Ridge) regularisation to prevent overfitting.
6. *Extreme Gradient Boosting with limited depth (XGBoost LD)*—Restricts the maximum depth of the decision trees in the ensemble to avoid overfitting.
7. *Extra Trees Regressor (ETR)*—Extra-trees differ from classic decision trees in the way they are built. When looking for the best split to separate a node’s samples into two groups, random splits are drawn for each randomly selected feature, and the best split among them is chosen.
8. *Light Gradient Boosting Machine (LightGBM)*—A gradient boosting framework that is optimised for speed and efficiency. Uses histogram-based decision tree learning, making it much faster than traditional boosting methods like XGBoost. Unlike XGBoost, which grows trees level-wise, LightGBM grows trees leaf-wise. LightGBM can lead to deeper trees and sometimes better accuracy but requires tuning to prevent overfitting.

To train tree-based models and SGD, we utilised FLAML, a lightweight Python 3.10 library designed for the efficient automation of machine learning and AI operations. It automatically selects the best model, optimises hyperparameters using cost-effective tuning, supports time budgeting to control how long AutoML runs, and adapts to dataset size and complexity, finding a good trade-off between accuracy and efficiency. The FLAML framework was configured with a time budget of 200 s per model for automated model parameter-tuning and optimisation. The coefficient $R^{2}$ was used as the evaluation metric for the regression task. Model performance was assessed using five-fold cross-validation, as specified by the chosen evaluation method and number of splits.

#### 2.4.3. Evaluating Metrics

To evaluate model performance, we used the following metrics:

**I.*** The coefficient of determination $R^{2}$*—A number between 0 and 1 that measures how well a statistical model predicts an outcome. $R^{2}$ can be interpreted as the proportion of variation in the dependent variable predicted by the statistical model. If the $R^{2}=1$—the model perfectly predicts unknown values.

$$R^{2}=1-\sum_{i}\frac{{\left(y_{i}-{\hat{y}}_{i}\right)}^{2}}{{\left(y_{i}-{\bar{y}}_{i}\right)}^{2}},$$

$y_{i}$—actual value, ${\bar{y}}_{i}$—average value, ${\hat{y}}_{i}$—predicted value.

**II.*** Mean Absolute Error* (MAE)—The average of the absolute value of the difference between actual and predicted values. The lower value is better.

$$\text{MAE}=\frac{1}{n}\sum_{i=1}^{n}\left|y_{i}-{\hat{y}}_{i}\right|.$$

**III.*** Mean Absolute Percentage Error* (MAPE)—Also known as mean absolute percentage deviation (MAPD), is a measure of prediction accuracy of a forecasting method in statistics. It usually expresses the accuracy as a ratio defined by the formula:

$$\text{MAPE}=\frac{1}{n}\sum_{i=1}^{n}\left|\frac{y_{i}-{\hat{y}}_{i}}{y_{i}}\right|.$$

A MAPE less than 5% is considered an indication that the forecast is acceptably accurate.

## 3. Results

### 3.1. Powder Characterisation

**Figure 5:**
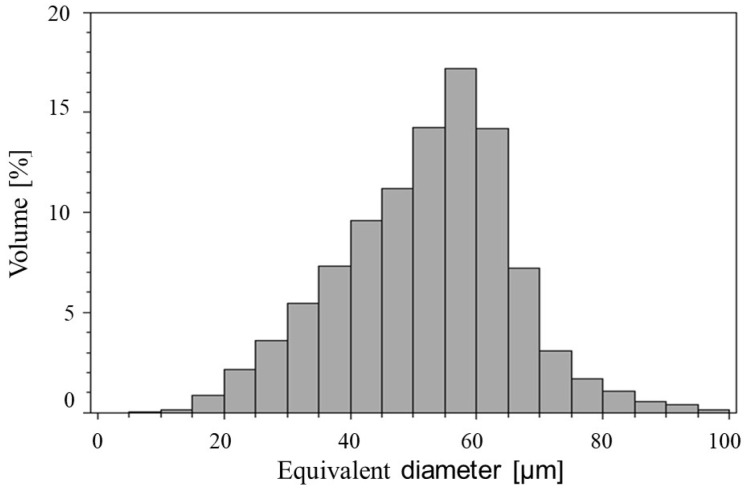
The H13 powder particle size distribution.

**Figure 6:**
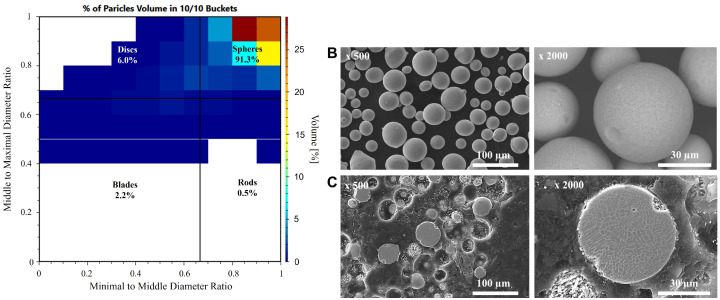
The H13 powder shape: Zingg chart with powder distribution measured by a parallel light beam analyser (**A**); observed under SEM microscope (**B**); observed under SEM microscope on a metallographic cross-section (**C**).

**Figure 7:**
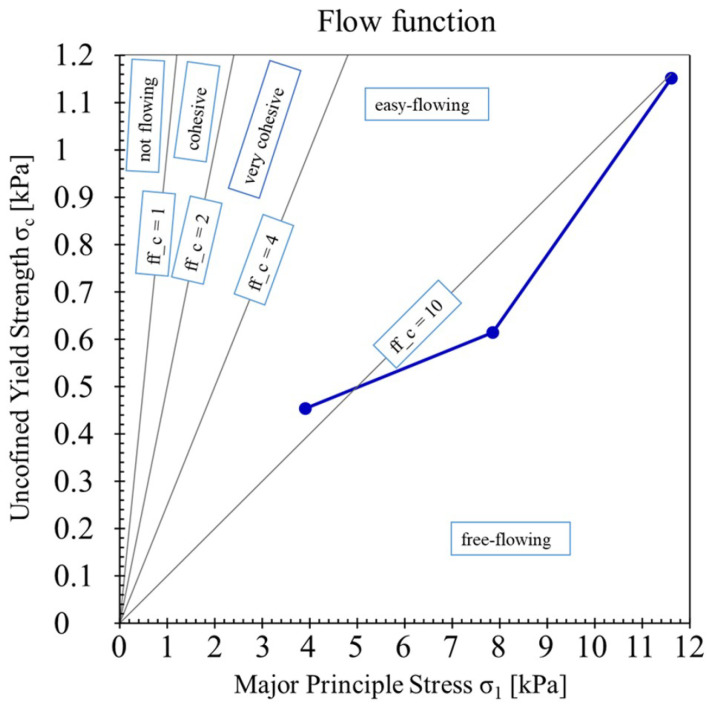
The flowability of the H13 powder measured using a powder rheometer.

Powder size analysis performed on a parallel light beam analyser confirmed that the average particle size of the H13 powder used in the research was Dv = 44.8 and Dn = 28.8 (Table 3 and Figure 5), consistent with the manufacturer’s stated range of 15–53 μm. The particle shape distribution analysis performed on a parallel light beam analyser (Figure 6A) showed that more than 91% volume of measured powders are spheres. Dominant spherical powder shape was also confirmed by SEM observations (Figure 6B,C). As seen in Figure 6C, some powder particles had pores that were closed within the particles. Furthermore, powder flowability test performed on a rheometer resulted in a flow coefficient ($ff_{c}$) above 4 (easy-flowing) or above 10 (free-flowing), regardless of the applied force (Figure 7). The powder’s microstructure (Figure 6C) has a cellular structure (dendrites) with clearly visible cell boundaries (interdendritic areas). The brighter cell boundaries may contain retained austenite, which is stable due to the increased carbon and alloying element content, which allows carbides to form in these areas [34,35,36].

### 3.2. Optimisation of the PBF-LB Process Parameters Without ML

**Figure 8:**
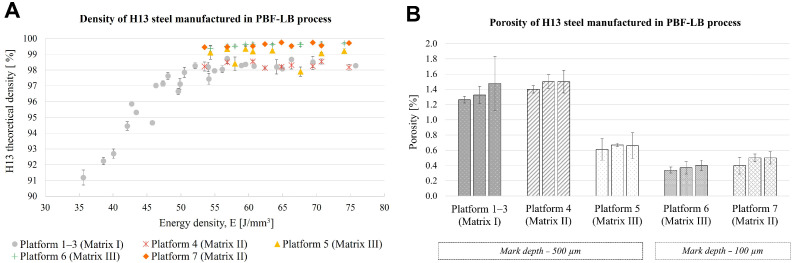
Relative density [%] of H13 steel samples manufactured on Platforms 1–7 (**A**) and Porosity of H13 steel samples manufactured using three sets of PBF-LB process parameters, which resulted in the lowest porosity value for each platform (**B**).

In the AM experimental study on H13 steel, the initial set of PBF-LB process parameters was derived from an established parameter set for H11 steel, provided by Aconity GmbH. These parameters included a laser power of “P” = 300 W, a scanning velocity of “V” = 1200 mm/s, a hatch distance of “H” = 80 μm, a laser spot size of “F” = 80 μm, a layer thickness “T” = 60 μm, and a preheating temperature for the building platform of “Temp” = 350 ^∘^C. Utilising these values, Matrix I (refer to Appendix A.2) was developed, consisting of 27 parameter sets with variations in P (increments of 50 W), V (increments of 100 mm/s), and H (increments of 10 μm). The parameters from Matrix I were tested across three distinct platforms (denoted as Platform 1–3), yielding porosity levels between 1.3% and 1.5% for three sets of PBF-LB parameters (see Figure 8). Following these initial trials, Matrix II was proposed, introducing a hatch distance H with steps of 5 μm, while retaining the three parameter sets identified from Platforms 1–3 that resulted in the lowest porosity. The parameter sets from Matrix II were utilised to produce samples on Platform 4, which maintained porosity levels similar to those observed in Matrix I, ranging from 1.4% to 1.5%.

Following the analysis of the best-performing parameter sets from Matrix II, Matrix III was developed. This new matrix adjusted the laser scanning speed, “V“, in increments of 50 mm/s. Out of the nine parameter sets tested in Matrix III, five successfully minimised porosity to a range between 0.6% and 0.8% on Platform 5. It’s noteworthy that four of these five top-performing sets had at least one sample excluded from the average calculation due to significant standard deviations observed in their respective series. Building on these results, Platform 6 was established using the parameter sets obtained from Matrix III, with the marking depth on the sample surface reduced from 500 μm to 100 μm. The objects produced from this platform exhibited porosity levels below 0.8%, with three parameter sets achieving an average porosity of 0.3–0.4%. Additionally, Platform 7 was made using Matrix II parameters, also with a modified depth of 100 μm. Platform 7 demonstrated a porosity range of 0.4% to 0.5%, reflecting a 1% decrease compared to the porosity achieved with Matrix II on Platform 4, which utilised a 500 μm depth.

### 3.3. ML Results

#### 3.3.1. ML Models Training Results

The PBF-LB process parameters and density results from platforms 1–7 were used for training ML models. Four models developed using FLAML (ETR, XGBoost LD, XGBoost, LightGBM), along with KRR, exhibited comparable CV $R^{2}$ values of approximately 0.96 for the training dataset, based on 5-fold cross-validation. Notably, the XGBoost model showcased the best performance on the testing data, achieving an $R^{2}$ of 0.977, a MAPE of 0.002, and a MAE of 0.017. The performance results for all models are summarised in Table 4.

**Figure 9:**
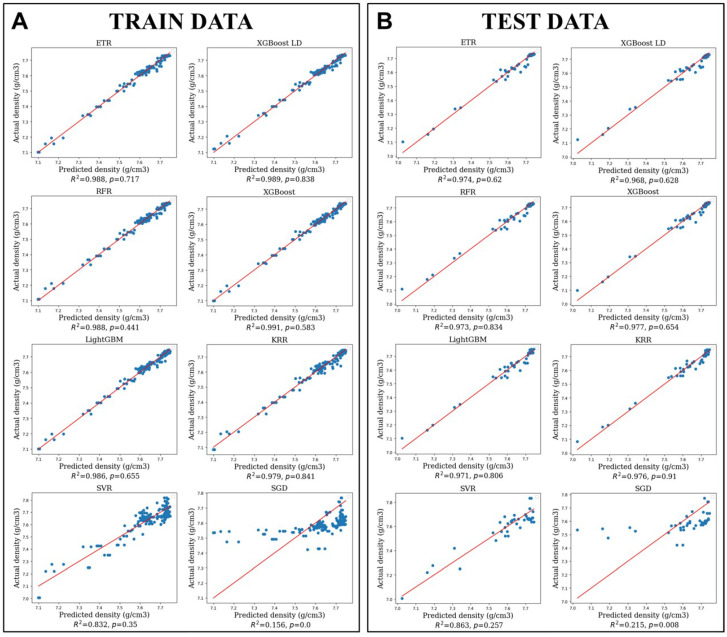
Train (**A**) and test (**B**) data of eight ML models used for porosity prediction of H13 steel.

Figure 9 illustrates the $R^{2}$ plots for all trained models (A—training data, B—test data). The plots clearly indicate that only the SGD model fell short in effectively addressing the task, while the other models demonstrated data points closely aligned with the 45-degree line. This alignment suggests that the predicted density values are in close proximity to the ground-truth values. Despite standard preprocessing and hyperparameter tuning, the linear nature of the SGD model limited its ability to capture the underlying relationships in the data, leading to unstable optimisation and a failure to converge. Almost all trained models (except for SVR) demonstrated similar results in terms of the mean and standard deviation of the predicted density values relative to the actual values on the test data, ranging from 0.22–0.26% and 0.22–0.29%, respectively. Only at a single point in the test dataset (Platform no.2—I.27, see Supplementary Material), all ML models’ predictions, except SVR, exhibit an absolute deviation between 1% and 1.4%. To evaluate the equality of distributions between the predicted and ground-truth density values, we employed a two-sided Wilcoxon test. The resulting p-value is greater than 0.05, indicating that there is no significant difference between the two distributions.

#### 3.3.2. Validation of the ML Models and PBF-LB Density Results from Platform 8

To determine the optimal settings for the PBF-LB process, we initially generated a grid of parameter values for “P” [W], “V” [mm/s], and “H” [μm] (see Table 5). For each combination of laser parameters, we predicted the sample density values using all the trained models. The coefficient of determination ($R^{2}$) obtained from cross-validation, along with the error metrics (MAE and MAPE), showed remarkable consistency across all models, except for the SVR model. The optimal laser parameters identified by the trained models, which yielded the maximum predicted density (“D” [g/cm^ 3^]), are summarised in Table 6. These PBF-LB process parameters were empirically validated by fabricating an additional platform (P8) that included, like for data collecting, three samples for each parameter set. The density of these manufactured samples was then measured using Archimedes’ method and compared with the ML models’ predicted values. After excluding the lowest-performing samples (one from each set of laser parameters), MAE and MAPE were calculated for the remaining samples, yielding values of 0.050 and 0.640, respectively. The MAE and MAPE values for the validation data of each model are also summarised in Table 6.

**Figure 10:**
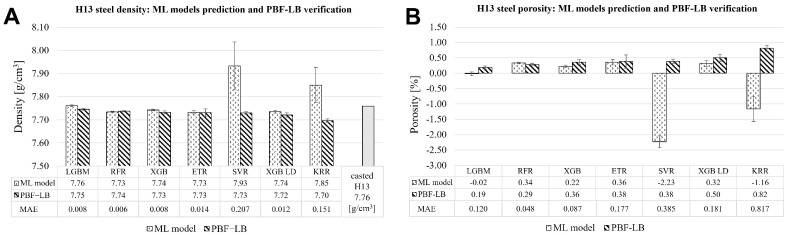
H13 steel density (**A**) and porosity (**B**) results—comparison of the ML models’ prediction and PBF-LB empirical verification. MAE error metrics values are presented for ML models. For PBF-LB, the fabrication error value is the standard deviation for 3 measurements.

Figure 10A illustrates the discrepancies in the density of H13 steel obtained from the parameters predicted by various machine learning models, including LightGBM/LGBM, RFR, XGBoost/XGB, SVR, XGBoost LD/XGB LD, and KRR, compared to the results from the PBF-LB experiments. Five of the seven models (LGBM, RFR, XGB, ETR, XGB LD) demonstrated a high degree of agreement with the predictions, yielding samples with densities exceeding 99.4% of the theoretical density of H13 steel. Utilising the parameters forecasted by four of the five most accurate models (LGBM, RFR, XGB, ETR), samples were produced with a theoretical density surpassing 99.6% of the specified density for H13 steel. The SVR model also facilitated the production of samples with densities above 99.6%; however, its predicted theoretical density for H13 steel was found to be 0.17 g/cm^3^ greater than the established theoretical value of 7.76 g/cm^3^. Conversely, the KRR model presented the lowest sample density achieved in the experiment, measuring 7.70 g/cm^3^, which resulted in a significant discrepancy of 0.11 g/cm^3^ between the predicted density and the PBF-LB experimental results. Figure 10B provides a visual representation of the differences in porosity, expressed as a percentage, computed using the ML models versus the values obtained from the PBF-LB experiments. It is important to note that although the actual porosity cannot be negative, two models (SVR and KRR) yielded such results, as their predicted density values exceeded the theoretical density for H13 steel. Continuous regression models such as KRR and SVR approximate the target function with a smooth mathematical relationship and lack built-in constraints on the range of output values. Consequently, when presented with a feature set that was not included in the training data, these models tend to extend the shape of the learned function, which can easily result in predictions that fall outside the observed values. To mitigate this issue, physical constraints can be integrated through custom loss functions or constrained regression techniques. In contrast, tree-based models such as RFR, LightGBM (LGBM), GBR, and ETR do not create a global smooth function. Instead, they generate predictions based on the average of target values within the terminal leaves. As a result, the predicted outputs are nearly always confined within the range of the training data, making these models more likely to produce physically plausible predictions.

The graph further indicates that the LightGBM model attained the lowest porosity value and the smallest deviation between the model predictions and the experimental outcomes. Consequently, the process parameters selected by the LightGBM model were utilised for subsequent experimental investigations, including the fabrication of heat treatment samples and a forging die fragment.

### 3.4. Microstructure and Mechanical Properties (Hardness) After Heat Treatments

**Figure 11:**
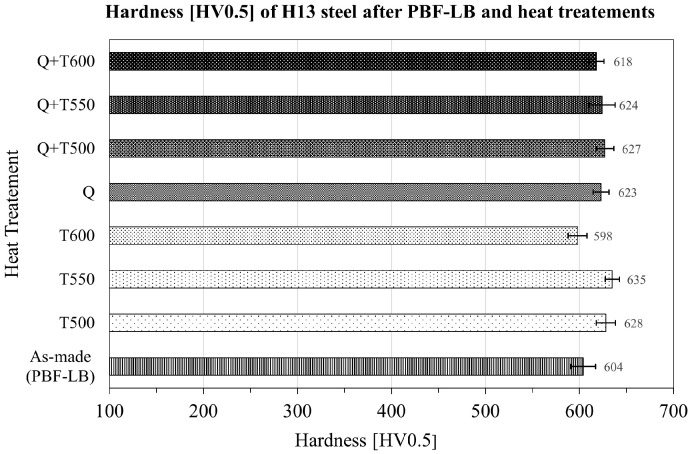
Hardness [HV0.5] of H13 steel samples fabricated in the PBF-LB process supported by ML optimisation after different heat treatments.

The hardness of H13 steel samples produced through PBF-LB fabrication, following parameter optimisation supported by machine learning and various heat treatments, is illustrated in Figure 11. The hardness of the as-fabricated PBF-LB sample, which was processed on a building platform preheated to 350 ° was 604 ± 13 HV0.5 (Figure 11). Following tempering at temperatures of 500 °C and 650 °C, the hardness of the material increased by approximately 30 HV0.5, reaching around 630 HV0.5. In contrast, tempering at 600 °C did not affect the material’s hardness. Additionally, both the quenching (Q) of H13 steel after the laser melting process and the combined quenching and tempering (Q + T) at temperatures of 500 °C, 550 °C, and 600 °C resulted in similar hardness level of 627, 624 and 618 HV0.5, respectively.

**Figure 12:**
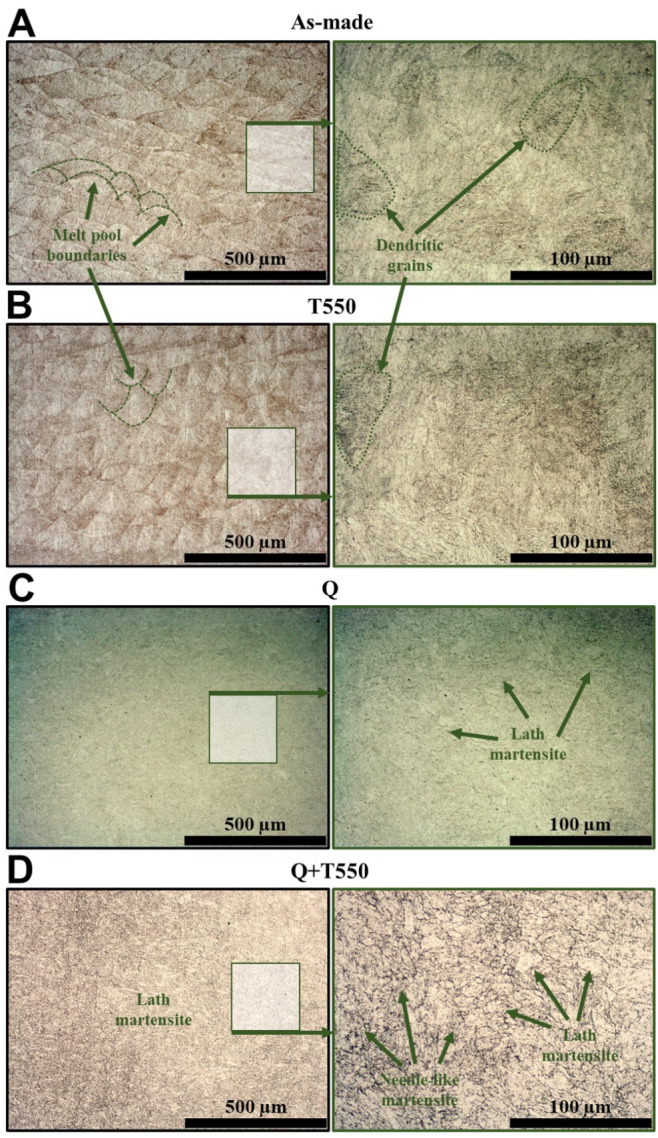
Microstructures of H13 steel samples fabricated in the PBF-LB process using the process parameters predicted by the ML - LightGBM model: without heat treatment/As-made (**A**), and after heat treatments: Tempering at 550 °C (**B**); Quenching (**C**); and Quenching + Tempering at 550 °C (**D**). Left side: marker 500 μm, right side: marker 100 μm.

The microstructure of H13 steel, fabricated through PBF-LB process using parameters predicted by the LightGBM machine learning model, is presented in Figure 12. The melt pool boundaries are distinctly observed in both the as-fabricated state (Figure 12A) and the tempered state at 550 °C (Figure 12B) following etching with a Nital reagent. The microstructure of both the as-fabricated and tempered samples exhibits a characteristic sub-grain cell structure, defined by a dendritic formation of long, thin grains that results from the rapid cooling during processing. When viewed perpendicularly to the axis of these dendritic grains, they appear as roughly circular shapes (Figure 12B). This cellular structure, consisting of a lath-like martensite core, was formed as a result of rapid cooling, whereas the interdendritic regions were enriched in alloying elements (Cr, Mo, V) and retained austenite, stabilised by local segregation during solidification [34,35,36,37,38,39]. Such features are consistent with previous observations for PBF-LB H13 steels by Deirmina et al. [34] and Li et al. [37], who reported that cyclic reheating between deposited layers promotes partial tempering and stabilisation of retained austenite.

The tempering process (Figure 12B) did not induce significant changes in the microstructure, as the melt pool boundaries typical of laser metal processing remained observable. However, small precipitates were detected at the grain boundaries post-tempering. Literature suggests that these precipitates may correspond to cementite or other phases enriched with alloying elements, e.g, fine $M_{7}$$C_{3}$ (Figure 12B, right side, higher magnification) [37,38] accompanied by the partial decomposition of retained austenite. This microstructure, partially characterised by fine, round cementite precipitates, is reminiscent of that observed following partial spheroidization heat treatment [39]. The extent of these microstructural changes was observed to increase with elevated tempering temperatures. The subtle differences in microstructure between the as-fabricated and tempered samples correspond to a modest increase in hardness, from approximately 600 HV0.5 to 630 HV0.5, consistent with the typical secondary hardening range for H13 steel [38,40]. Quenching of the samples following the PBF-LB process led to the elimination of melt pool boundaries and columnar dendritic grains (Figure 12C). The resultant microstructure was comprised of lath martensite packets, as well as finer needle-like and lamellar martensite (Figure 12C, right side, higher magnification). The microstructure resulting from the quenching and tempering (denoted as Q + T550) displayed similarities to that of the as-fabricated sample tempered at 550 °C (Figure 12B); however, the dendritic grain boundaries remained concealed, despite prolonged etching times.

### 3.5. Forging Die

**Figure 13:**
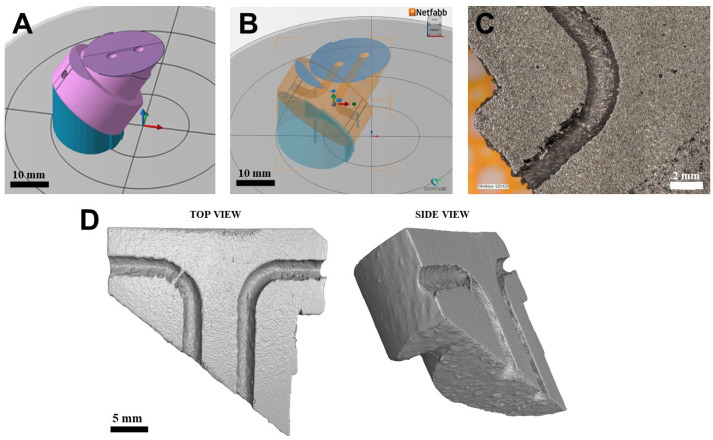
A part of the forging die fabricated using the PBF-LB process with the process parameters predicted by the LightGBM ML model; CAD model (**A**), CAD model with visible internal channels (**B**), metallographic cross-section (**C**) and XCT reconstruction (**D**).

A section of the forging die, produced in the PBF-LB process using parameters optimised by LightGBM model, is depicted in Figure 13. This section was fabricated on support structures inclined 60° to the building platform (illustrated in Figure 13A) and incorporates two conformal channels (shown in Figure 13B). The metallographic cross-section revealed that the channels exhibited some irregular surfaces, aligned parallel to the building platform. Notably, these channels are pass-through, ensuring that no powder was trapped inside. Furthermore, Archimedes’ measurements confirmed that the porosity is below 0.5%. The X-ray Computed Tomography (XCT) cross-section reconstruction of the forging die is presented in Figure 13.

## 4. Discussion

### 4.1. PBF-LB Processing

To obtain functional objects with the required mechanical properties in the PBF-LB process, it is essential to minimise defects such as porosity and cracks [41,42]. This can be achieved by using high-quality feedstock materials and carefully selected parameters that ensure the continuity of the molten metal pool [43,44]. Additionally, internal stresses and non-equilibrium or non-compliant with ASTM/ISO standards microstructures resulting from the PBF-LB process can be mitigated through heat treatments [45,46,47].

In our research, we utilised H13 steel powders, which had a wide range of particle sizes, from around 3 μm to 96 μm (Figure 5). Furthermore, H13 steel powder was characterised by very high sphericity—more than 91% of powder particles in measured sample were spheres (Figure 6A) with a high flow coefficient—$ff_{c}>4$ (Figure 7). Moreover, used in our research steel feedstock was characterised by minimal internal porosity seen only within individual particles (Figure 6B). This choice of materials ensured that the porosity observed during the parameter selection stage of the PBF-LB process was related to the process itself and its parameters, rather than the quality of the feedstock. It is essential to note that H13 steel is recognised as challenging to weld and prone to cracking due to the high stresses it generates during production, which are attributed to its chemical composition [48]. To address this issue, we carried out the PBF-LB process at an elevated build plate temperature of up to 350 °C, a condition that has not been previously reported for H13 steel. PBF-LB processes conducted at a building platform heated above 200–250 °C are rarely documented in the literature, as most commercially available devices do not support heating the building plate beyond this temperature range.

The traditional methodology for selecting PBF-LB process parameters typically involves modifying a set of parameters developed by the device manufacturer through a trial-and-error process. Another method is to create your own matrices with PBF-LB parameter sets and material characterisation of fabricated samples one by one until the required microstructure is obtained with a slight change of parameters during optimisation [49,50]. In our study, the initial set of parameters was adapted from the set of H11 steel parameters distributed by Aconity GmbH (Aconity device manufacturer). This set, prepared for H11 steel, is expected to result in porosity below 1% when used by Aconity GmbH powder supplier. Based on the parameters set for H11 steel, we prepared for H13 steel process optimisation nine sets of parameters (Matrix I—Appendix A.2), each adjusting three key variables of the PBF-LB process: laser power “P” [W], scanning speed “V” [mm/s], and distance between scan lines “H” [mm]. Using these three arrays, we fabricated seven platforms with 189 test samples. Each array produced three samples with a specific set of parameters, resulting in porosity levels below 0.6% for most samples from platforms 5, 6, and 7, where values below 1% are generally acceptable in industrial applications. Notably, the lowest porosity values, below 0.4%, were achieved not by modifying the PBF-LB process parameters, but by adjusting the marking depth for relatively small (10 mm diameter and 10 mm height) test samples. This marking depth was reduced to 100 micrometres for platforms 6 and 7, compared to 500 micrometres for platforms 1–5.

The challenge of identifying a single “best” set of PBF-LB process parameters for producing H13 steel prompted us to test eight selected ML models using the collected data.

### 4.2. ML Prediction Results

Despite the significant number of publications in recent years focused on using ML to optimise laser processing parameters for additive manufacturing of various metals, there have been no ML-based studies specifically addressing laser parameter optimisation for H13 steel. Yonehara et al. [51] optimised laser power, scan speed, and hatch spacing through a series of experiments that tested various parameter combinations, achieving a density of 99.9% at 400 W ($R^{2}$ not reported; experimental validation) using a trial-and-error approach without incorporating ML methods. In another study [52], single-track PBF-LB/M experiments were conducted to establish a parameter window that would yield defect-free parts, identifying continuous tracks with depth-to-width ratios of 0.4–0.6 as optimal. Several key machine learning techniques have been reported for predicting porosity and density in different steels, including: 1. GPR for 17-4 PH [53]; 2. SVM and ANN for 316L SS [54]; 3. Ensemble Tree Algorithms (such as RFR and XGBoost) for 316L SS [55,56]. In this work, we evaluated the performance of models, including KRR, SVR, SGD, RFR, XGBoost, XGBoost LD, ETR, and LightGBM, to predict the theoretical density “D” [g/cm^3^] based on selected PBF-LB process parameters. Initially, we conducted trials with SVR and KRR, as these methods are frequently cited in studies related to predicting the physical properties of samples produced via PBF-LB for various alloys [10,54,56] and have demonstrated relatively high prediction accuracy. Subsequently, we trained various ensemble models, which offer the primary advantages of increased accuracy and robustness compared to the usage of a single model [32,57].

Feature analysis revealed that H13 steel theoretical density was strongly correlated with volumetric “E” [J/mm^3^] and linear “LE” [J/mm^3^] energy densities. Other parameters, such as laser power “P” [W] and the depth of sample marking “Mark depth” (100 μm or 500 μm), also showed notable effects. The absence of high inter-feature correlations suggested that non-linear models could better capture the underlying dependencies. Following preprocessing, including encoding categorical variables and normalising feature ranges, the trained models were applied to predict optimal process parameters for the PBF-LB process. Its experimental validation (Platform No. 8) confirmed the effectiveness of the predictive framework, as the models successfully identified laser parameter combinations that yielded a high-density (mostly above 99.6% of the 7.76 g/cm^3^ theoretical density of H13 steel) H13 steel sample. The comparative analysis of the trained models demonstrated that ETR, XGB, XGB LD, RF, LGBM and KRR achieved similar performance on the training dataset (CV $R^{2}\approx 0.96$). However, XGBoost proved to be the most effective on the testing data, yielding $R^{2}$ = 0.977, MAPE = 0.017, and MAE = 0.02, indicating excellent predictive accuracy. Although a few test points exhibited larger deviations (∼1%) between predicted and experimental values, the majority of predictions showed minimal errors with a mean absolute difference of 0.22% and a standard deviation of 0.27%.

A clear distinction arises when comparing the statistical performance of different models (see Table 4 and Figure 9) with their experimental reliability (see Table 6 and Figure 10). To validate the ML results, optimal laser parameters corresponding to the maximum predicted density were selected for each trained model, as shown in Table 6. While the XGBoost model maintained high calculated predictive accuracy, the LightGBM model demonstrated greater reliability and generalizability in practical applications (PBF-LB processing). Specifically, the predicted density values from LightGBM showed the closest agreement with both the theoretical density of H13 steel and the experimentally measured densities of the PBF-LB samples, with deviations not exceeding 0.2% (refer to Figure 10A,B).

In contrast, although the KRR model exhibited a high cross-validation $R^{2}$ and competitive MSE and MAPE values, it predicted maximum density values that substantially exceeded the theoretical density of H13 steel. This overestimation indicates a limited physical consistency of the model, a finding confirmed by validation experiments (see Figure 10A,B). The SVR model showed the weakest performance from the outset, with a CV $R^{2}$ of 0.785, MAPE of 0.007, and MAE of 0.052, leading to unrealistically high density predictions beyond the theoretical limit. Consequently, during Archimedes’ density validation, measured densities deviated from the predicted values by 2.6%, highlighting the model’s inadequacy for reliable predictions.

By comparing our results with previously reported data for other materials, we conclude that our trained models predict sample porosity with comparable or superior accuracy. Studies on 316L stainless steel have demonstrated that classical ML algorithms, such as KNN, SVM, logistic regression, and ensemble tree-based methods, can achieve accuracies of up to 96%, with $R^{2}$ values ranging from 0.95 to 0.99 [54,55,56]. Similarly, linear regression, ANN, KNN, and SVM have been employed to predict the density of 316L stainless steel. The best-performing ANN model achieving $R^{2}$ = 0.95 and MAE of 3.56 [54]. Tao Shen et al. [58] reported that a multilayer perceptron (MLP) with three hidden layers and feature engineering techniques, achieved an $R^{2}$ of 0.954 when predicting the relative density of Ti-6Al-4V alloy parts manufactured using the PBF-LB process. In contrast, another study that applied seven ML algorithms to 316L stainless steel parts processed by PBF-LB reported lower accuracy, with the Gradient Boosting Regressor (GBR) B algorithm achieving only $R^{2}$ = 0.6296 and MAE = 0.4665. Regarding Al-10Si-Mg alloys fabricated by PBF-LB, predictive modelling of porosity using an adaptive-network-based fuzzy inference system achieved an $R^{2}$ of 0.97 [59]. Table 7 summarises the ML porosity prediction results from current and other published studies.

In many previous studies, training data were collected from multiple literature sources with different measurement protocols and machine conditions, which may introduce inconsistent input–output relationships and degrade model performance. In addition, some commonly used ML techniques assume linear relationships and therefore fail to capture the complex nonlinear interactions inherent to the PBF process, while limited experimental diversity can further restrict model generalization. In contrast, in this study, the data were obtained under consistent experimental conditions across a sufficiently wide range of laser parameters, the selected ML models are capable of approximating complex nonlinear dependencies, and the optimal parameter sets were validated through an independent experimental campaign. In addition, the standard set of features—laser power “P” (W), scanning speed “V” (mm/s), hatch distance “H” (μm), and layer thickness “T” (μm)—was expanded to include volumetric energy density “E” (J/mm^3^), linear energy density “LE” (J/mm^2^), the sample position on the build platform (denoted on each sample by a number on the top surface and referred to as “Placement”), and the sample marking depth (“Mark depth”). These additional features demonstrated a high degree of correlation with the target function.

### 4.3. Heat Treatments and Hardness

Steels used for forging tools must have various properties at an appropriately high level to cope with the problematic conditions present during the forging process. One of the requirements for these steels is the appropriate hardness, indicating the high strength of the material. The elevated build platform temperature of 350 °C in our study served as an in-situ heat treatment, partially relieving residual stresses and significantly reducing the risk of cracking in this high-hardenability tool steel. Moderate preheating of the building platform, in the range of 200–400 °C, during PBF-LB fabrication of H13 steel effectively promotes stress relaxation and stabilises melt pool geometry, resulting in a more uniform material [61,62]. In contrast, excessive preheating above 450 °C can suppress martensitic transformations and promote extensive tempering of the microstructure. The preheating level used in this work, therefore, provides a favorable compromise between build quality, dimensional stability, and microstructural control [63]. To ensure the high strength and homogeneity of the tested steels throughout their volume, the H13 steel samples were subjected to various heat treatment processes. Moreover, heat treatment of steel after the additive manufacturing process removes unwanted internal stresses created during this process, which may be responsible for tool cracks in the future [36]. The hardness of the As-made samples, processed using the LightGBM model-predicted parameters, on the building platform preheated to 350 °C, was 604±13 HV0.5, which is similar to that of tools used in the forging industry, indicating that the optimal fabrication parameters have been selected. During the PBF-LB fabrication process, the material solidifies from the melt at very high cooling rates, beginning just below the liquidus temperature. Then, the material is cyclically exposed to elevated temperatures, which results from the application of successive layers of material. Depending on the process parameters, the initial thermal cycles for H13 steel cause the material to recrystallise due to fast cooling from the austenitization temperature. Subsequent cycles lead to tempering. As a result of these thermal cycles, the material within the sample ultimately becomes tempered steel. However, because the amount of material used in subsequent runs is small, the tempering process is brief and does not occur fully. Consequently, tempering of laser-processed material increased the hardness of H13 steel, as the tempering could proceed more fully due to the effects of secondary hardening. Tempering at 500 °C was likely cooler than the previous tempering temperatures of the material during the PBF-LB process. Therefore, it did not raise the temperature compared to the material in its as-made state. The hardness of the PBF-LB fabricated material after the quenching process is approximately 625 HV (50–51 HRC), as shown in Figure 11. This value is roughly 10 HV lower than the typical range of 51–53 HRC (635–670 HV) reported for wrought H13 steel that has undergone oil quenching from 1050 °C [40]. This discrepancy can be attributed to the lath martensite morphology illustrated in Figure 12C. The martensite present in our PBF-LB samples is notably finer and more densely packed than that in conventionally processed H13, due to the exceptional cooling rates ($10^{5}$ K/s) characteristic of laser-based additive manufacturing. In contrast, wrought H13 experiences significantly slower cooling rates during traditional forging and heat treatment, leading to coarser martensitic lath structures that may accommodate higher local dislocation densities; however, verifying this hypothesis would require a more thorough quantitative microstructural analysis. While the refined martensite morphology provides enhanced toughness and ductility relative to its coarser conventional counterparts, it also yields lower hardness values. Moreover, the retained austenite observed in the interdendritic regions (enriched in Cr, Mo, and V) serves as a softer phase interspersed within the martensitic matrix. Although the hardness is slightly lower than that of conventionally processed H13, it remains entirely suitable for tool applications, especially where thermal fatigue resistance, ductility, and a refined grain structure confer superior service performance.

### 4.4. Limitations and Future Work

While the results of the present study are promising, several limitations must be acknowledged. Firstly, the dataset utilised is relatively small, which may hinder the generalisation capability of the trained models and increase their susceptibility to noise or outliers. Secondly, the process variables examined were confined to a predefined set of PBF-LB parameters, without considering other potentially influential factors, such as powder morphology, chemical composition, or environmental conditions, for example, gas flow or oxygen content during the process. This limitation may restrict the model’s ability to fully capture the complexity of process–structure relationships. Additionally, the experiments were conducted using a single AM process system with specific hardware configurations and control strategies. Consequently, directly transferring trained models to other PBF-LB machines may lead to diminished predictive performance due to machine-to-machine variability. Variations in recoating mechanisms, oxygen content, and gas flow intensity further challenge model portability.

Future research should focus on expanding the dataset to include a broader range of process parameters and build conditions, thereby enhancing the models’ robustness. Including data from multiple AM process systems would facilitate systematic evaluations of model transferability and contribute to the development of more generalizable predictive frameworks. Another promising avenue is the integration of physics-informed constraints to mitigate non-physical predictions and improve extrapolation capabilities. Finally, using adaptive or transfer learning strategies can adjust an existing model to different machines or materials by reusing prior training, enabling efficient recalibration with only a small amount of new experimental data. Results of this study provide a basis for future research to utilise the developed ML models to predict parameters of the PBF-LB process for other alloy groups, such as new copper and titanium alloys.

## 5. Conclusions

Our research results confirm that the implemented ML framework, based on automated hyperparameter optimisation using the FLAML library, effectively captures complex non-linear relationships between PBF-LB process parameters and relative density. Our research demonstrates that even with a limited experimental dataset (n=189), properly tuned ensemble models can provide robust and generalisable predictions. The best-performing model, XGBoost, achieved an $R^{2}$ value of 0.977, MAPE = 0.002, and MAE = 0.017, surpassing most results reported for similar alloys in the literature. This validates the suitability of automated ML approaches for efficient parameter screening and process window identification prior to experimental validation, thereby substantially reducing the number of physical trials required.

The LightGBM model provided the most reliable and experimentally validated prediction, yielding additional manufactured samples with a relative density above 99.6% of the theoretical value (7.76 g/cm^3^). These parameters also enabled successful fabrication of a forging die segment with internal conformal channels and porosity below 0.5%, demonstrating the feasibility of ML-guided optimisation for industrial-scale PBF-LB production.

Microstructural analyses of samples fabricated with the LightGBM model predicted PBF-LB parameters confirmed a fine cellular martensitic structure typical of laser-processed H13 steel, with limited retained austenite. After tempering at 550 °C, hardness increased slightly to approximately 630 HV0.5, indicating partial secondary hardening without significant grain coarsening. Post-process heat treatment, involving re-quenching and tempering, reduced microstructural heterogeneity and improved homogeneity, although the hardness remained unchanged at a level typical of conventionally processed H13 steel.

## Figures and Tables

**Table 1:** PBF-LB parameters used for the sample’s fabrication for training and testing ML models.

PBF-LB Parameters Modified During the Study		
**Parameter**	**Range**	**Step**
Laser power “P” [W]	250–350 W	50 W
Scanning speed “V” [mm/s]	1050–1300 mm/s	50 mm/s
Distance between scanning vectors “H” [μm]	65–90 μm	5 μm or 10 μm
Depth of sample marking “Mark depth” [μm] (Figure 1C)	100 μm or 500 μm	-
**PBF-LB Parameters Constant During the Study**		
**Parameter**		**Value**
Layer thickness “T” [μm] *		60 μm
Building platform preheating temperature “Temp.” [°C] *		350°C
Laser spot “F” [μm] *		80 μm
Scanning strategy *		Alternating; stripe size 5 mm
Sample placement on building platform *		1–27
Rotation angle between layers **		67°

* Information removed from ML data during the preprocessing stage. ** Information removed/not used in ML dataset.

**Table 2:** Heat treatments performed in the research.

	Quenching	Tempering
	**Temperature [°C]/Time [min]/**	**Temperature [°C]/Time [min]/**
	**Cooling Medium**	**Cooling Medium**
As-made (PBF-LB)	—	—
T500	—	500 °C/90 min/Air
T550	—	550 °C/90 min/Air
T600	—	600 °C/90 min/Air
Q	1050 °C/ 20 min/Oil	—
Q + T500	1050 °C/20 min/Oil	500 °C/90 min/Air
Q + T550	1050 °C/20 min/Oil	500 °C/90 min/Air
Q + T600	1050 °C/20 min/Oil	600 °C/90 min/Air

**Table 3:** The H13 powder particle size measurement results.

Number of particles in the sample		97,898
Mean Dv (Volume) [μm]		44.8
Mean Dn (Quantitative) [μm]		28.8
Minimum D [μm]		3.2
Maximum D [μm]		95.5
Volumetric (Dv) and Quantitative (Dn) percentiles		
Dv [μm]	Dn [μm]	
10	26.4	14.7
50	45.0	26.4
90	62.4	47.4

**Table 4:** ML models’ performance ranked according to the highest $R^{2}$ test coefficient.

Model	CV $R^{2}$	$R^{2}$ Train	$R^{2}$ Test	MAPE Test	MAE Test
ETR	0.969	0.988	0.974	0.002	0.017
XGBoost LD	0.968	0.989	0.969	0.003	0.020
RFR	0.965	0.988	0.973	0.003	0.019
XGBoost	0.964	0.991	0.977	0.002	0.017
LightGBM	0.963	0.986	0.971	0.003	0.020
KRR	0.957	0.979	0.976	0.003	0.020
SVR	0.785	0.832	0.863	0.007	0.052
SGD *	-	-	-	-	-

* The SGD model did not converge to optimal parameters in the FLAML framework.

**Table 5:** PBF-LB parameters employed in hyperparameter optimisation using predictions of the best ML.

Parameter	Range/Values	Step
Laser power “P” [W]	250–350 W	5 W
Scanning speed “V” [mm/s]	1050–1300 mm/s	10 mm/s
Distance between scanning vectors “H” [μm]	65–90 μm	1 μm
The position of the samples on the platform	1–27	1
The depth of sample marking	0.1 mm, 0.5 mm	-

**Table 6:** Selected sets of PBF-LB process parameters for each ML model for PBF-LB experimental verification.

ML Model	“P” [W]	“V” [mm/s]	“H” [μm]	“D” [g/cm^3^]	“E” * [J/mm^3^]	MAE [g/cm^3^]	MAPE [%]
ETR	350	1200	78	7.732	62	0.014	0.181
XGBoost	350	1220	81	7.735	60	0.012	0.151
LD							
RFR	350	1240	70	7.734	67	0.006	0.078
XGBoost	350	1100	76	7.743	70	0.008	0.099
LightGBM	330	1070	71	7.761	72	0.008	0.099
KRR	305	1300	66	7.851	59	0.151	1.961
SVR	300	1050	65	7.937	73	0.207	2.678
SGD **	-	-	-	-	-	-	-

* Energy density “E” [J/mm^3^] was calculated based on PBF-LB parameters: variable value of “P” [W], “V” [mm/s], “H” [μm] and constant “T” = 60 μm. ** SGD was removed for prediction due to not converging on optimal parameters in the FLAML framework.

**Table 7:** ML porosity prediction in PBF-LB processed alloys.

Source	Alloy	Model	$R^{2}$
Barrionuevo G.O. et al. [55]	316L SS	GBR	0.6296
Gor M. et al. [54]	316L SS	ANN	0.95
Eshkabilov E. et al. [56]	316L SS	SVM	0.98
Tao Shen et al. [58]	Ti-6Al-4V	MLP with FE	0.954
Babakan A.M. et al. [59]	Al-10Si-Mg	Adaptive-network-based fuzzy inference system model	0.97
Amar et al. [60]	316L SS	Second-order polynomial regression	0.94
This study	H13 Tool Steel	ETR	0.969
		XGBoost LD	0.968
		RFR	0.965
		XGBoost	0.964
		LightGBM	0.963
		KRR	0.957
		SVR	0.785

**Figure A1:**
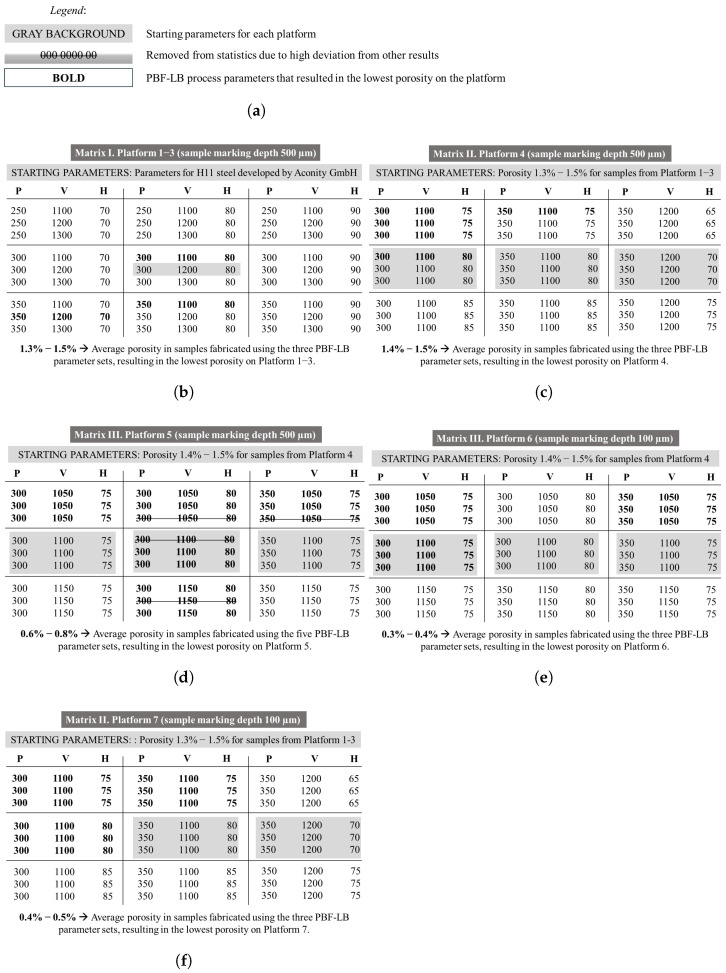
Matrices of PBF-LB Parameters Used for Classic and ML-Supported Optimisation.

## Data Availability

The original contributions presented in this study are included in the article/Supplementary Material. Further inquiries can be directed to the corresponding author.

## Supplementary Materials

The following supporting information can be downloaded at https://www.mdpi.com/article/10.3390/ma19010210/s1. Excel spreadsheet with PBF-LB process parameters and ML data (File: KKosarava_H13_PBF-LB.xlsx).

## Appendix A

### Appendix A.1. Chemical Composition of H13 Steel Powder

See Table A1.

**Table A1:** Chemical composition of H13 steel powder.

Chemical Composition		
**Element**	**Content [wt. %]—Höganäs**	**Content [wt. %]—MRC**
Cr	5.2	4.8
Mo	1.5	1.5
V	1.0	1.0
Si	1.0	1.6
C	0.35	-
Mn	0.3	0.3
O	0.05	-
Fe	Balance	Balance

## References

[1] Totten G.E. (2006). Steel Heat Treatment Metallurgy and Technologies. Steel Treatment Handbook.

[2] Davis J.R. (1995). ASM Specialty Handbook: Tool Materials.

[3] Kou S. (2002). Work-Hardened Materials. Welding Metallurgy.

[4] Lippold J.C., Kotecki D.J. (2014). Welding Metallurgy and Weldability.

[5] (2021). Additive Manufacturing—General Principles—Fundamentals and Vocabulary.

[6] Huang X., Kang N., Wang Q., El Mansori M., Guittonneau F. (2025). Wear of directed energy deposited H13 steel as a function of its graded microstructure. Tribol. Int..

[7] Tanvir A.N.M., Ahsan M.R.U., Seo G., Bates B., Lee C., Liaw P.K., Noakes M., Nycz A., Ji C., Kim D.B. (2021). Phase stability and mechanical properties of wire + arc additively manufactured H13 tool steel at elevated temperatures. J. Mater. Sci. Technol..

[8] Nandwana P., Goldsby D., Siddel D., Kannan R., Sears J. (2021). Binder Jet Printing of H13 Tool Steel.

[9] Szcześniak K., Pawlak A., Dybała B., Kras A. (2024). The importance of adjusting the processing parameters for the resulting material density of PBF-LB AISI 316L lattice structures. Arch. Civ. Mech. Eng..

[10] Liu J., Ye J., Silva Izquierdo D., Vinel A., Shamsaei N., Shao S. (2023). A review of machine learning techniques for process and performance optimization in laser beam powder bed fusion additive manufacturing. J. Intell. Manuf..

[11] Awd M., Saeed L., Walther F. (2023). A review on the enhancement of failure mechanisms modeling in additively manufactured structures by machine learning. Eng. Fail. Anal..

[12] Wong R., Tran A., Dovgyy B., Maldonado C.S., Pham M.S. (2025). Critical statistical assessment of data in metal additive manufacturing. Mater. Des..

[13] Maitra V., Arrasmith C., Shi J. (2024). Introducing explainable artificial intelligence to property prediction in metal additive manufacturing. Manuf. Lett..

[14] Luo Q., Shimanek J.D., Simpson T.W., Beese A.M. (2024). An Image-Based Transfer Learning Approach for Using In Situ Processing Data to Predict Laser Powder Bed Fusion Additively Manufactured Ti-6Al-4V Mechanical Properties. 3D Print. Addit. Manuf..

[15] Wang H., Li B., Gong J., Xuan F.Z. (2023). Machine learning-based fatigue life prediction of metal materials: Perspectives of physics-informed and data-driven hybrid methods. Eng. Fract. Mech..

[16] Hamada A., Elyamny S., Abd-Elaziem W., Elkatatny S., Darwish M.A., Sebaey T.A., Järvenpää A., Vineesh K.P., Elsheikh A.H. (2025). Advancing fatigue life prediction with machine learning: A review. Mater. Today Commun..

[17] Zhan Z., Li H. (2021). Machine learning based fatigue life prediction with effects of additive manufacturing process parameters for printed SS 316L. Int. J. Fatigue.

[18] Banerjee S., Thapliyal S., Agilan M., Dineshraj S., Bajargan G., Sigatapu S. (2025). A Machine Learning-Based Model for Multiple Material Density Prediction Developed by Powder Bed Fusion Additive Manufacturing. J. Mater. Eng. Perform..

[19] Akbari P., Zamani M., Mostafaei A. (2024). Machine learning prediction of mechanical properties in metal additive manufacturing. Addit. Manuf..

[20] Riensche A.R., Bevans B.D., King G., Krishnan A., Cole K.D., Rao P. (2024). Predicting meltpool depth and primary dendritic arm spacing in laser powder bed fusion additive manufacturing using physics-based machine learning. Mater. Des..

[21] Akbari P., Ogoke F., Kao N.Y., Meidani K., Yeh C.Y., Lee W., Farimani A.B. (2022). MeltpoolNet: Melt pool characteristic prediction in Metal Additive Manufacturing using machine learning. Addit. Manuf..

[22] Ghayoomi Mohammadi M., Mahmoud D., Elbestawi M. (2021). On the application of machine learning for defect detection in L-PBF additive manufacturing. Opt. Laser Technol..

[23] Yang M., Rezaei A., Vlasea M. (2025). Process screening in additive manufacturing: Detection of keyhole mode using surface topography and machine learning. Addit. Manuf. Lett..

[24] Dey S., Lyu Z., Mahalle G., Achouri A., Mamun A.A. (2022). Application of Deep Learning models to characterize manufacturing defects in additive manufactured components. Procedia Struct. Integr..

[25] Kanko J.A., Sibley A.P., Fraser J.M. (2016). In situ morphology-based defect detection of selective laser melting through inline coherent imaging. J. Mater. Process. Technol..

[26] Yuan M., Cao Y., Karamchedu S., Hosseini S., Yao Y., Berglund J., Liu L., Nyborg L. (2022). Characteristics of a modified H13 hot-work tool steel fabricated by means of laser beam powder bed fusion. Mater. Sci. Eng. A.

[27] Ghoncheh M.H., Shahriari A., Birbilis N., Mohammadi M. (2024). Process-microstructure-corrosion of additively manufactured steels: A review. Crit. Rev. Solid State Mater. Sci..

[28] Mukherjee T., Elmer J., Wei H., Lienert T., Zhang W., Kou S., DebRoy T. (2023). Control of grain structure, phases, and defects in additive manufacturing of high-performance metallic components. Prog. Mater. Sci..

[29] Ali H., Ma L., Ghadbeigi H., Mumtaz K. (2017). In-situ residual stress reduction, martensitic decomposition and mechanical properties enhancement through high temperature powder bed pre-heating of Selective Laser Melted Ti6Al4V. Mater. Sci. Eng. A.

[30] Cong D., Zhou H., Yang M., Zhang Z., Zhang P., Meng C., Wang C. (2013). The mechanical properties of H13 die steel repaired by a biomimetic laser technique. Opt. Laser Technol..

[31] Massey F.J. (1951). The Kolmogorov-Smirnov Test for Goodness of Fit. J. Am. Stat. Assoc..

[32] Smola A.J., Schölkopf B. (2004). A tutorial on support vector regression. Stat. Comput..

[33] Welling M. (2011). A First Encounter with Machine Learning. https://www.studocu.com/en-za/document/university-of-south-africa/machine-learning/a-first-encounter-with-machine-learning-machine-learning-book-max-welling-2011/14883427.

[34] Deirmina F., Peghini N., AlMangour B., Grzesiak D., Pellizzari M. (2019). Heat treatment and properties of a hot work tool steel fabricated by additive manufacturing. Mater. Sci. Eng. A.

[35] He Y., Zhong M., Beuth J., Webler B. (2020). A study of microstructure and cracking behavior of H13 tool steel produced by laser powder bed fusion using single-tracks, multi-track pads, and 3D cubes. J. Mater. Process. Technol..

[36] Haghdadi N., Laleh M., Moyle M., Primig S. (2021). Additive manufacturing of steels: A review of achievements and challenges. J. Mater. Sci..

[37] Li S., Yang S., Zhao Y., Dong Y., Wang Z. (2023). 2 GPa H13 steels fabricated by laser powder bed fusion and tempering: Microstructure, tensile property and strengthening mechanism. Mater. Sci. Eng. A.

[38] Bae K., Moon H., Park Y., Jo I., Lee J. (2022). Influence of Tempering Temperature and Time on Microstructure and Mechanical Properties of Additively Manufactured H13 Tool Steel. Materials.

[39] Soleimani M., Mirzadeh H., Dehghanian C. (2021). Effects of spheroidization heat treatment and intercritical annealing on mechanical properties and corrosion resistance of medium carbon dual phase steel. Mater. Chem. Phys..

[40] Yan G., Huang X., Wang Y., Qin X., Yang M., Chu Z., Jin K. (2010). Effects of heat treatment on mechanical properties of h13 steel. Met. Sci. Heat Treat..

[41] Kaletsch A., Qin S., Herzog S., Broeckmann C. (2021). Influence of high initial porosity introduced by laser powder bed fusion on the fatigue strength of Inconel 718 after post-processing with hot isostatic pressing. Addit. Manuf..

[42] Ledwaba T., Steenkamp C., Chmielewska-Wysocka A., Wysocki B., du Plessis A. (2025). Development of AI crack segmentation models for additive manufacturing. Tomogr. Mater. Struct..

[43] Haferkamp L., Liechti S., Spierings A., Wegener K. (2021). Effect of bimodal powder blends on part density and melt pool fluctuation in laser powder bed fusion. Prog. Addit. Manuf..

[44] Spurek M.A., Haferkamp L., Weiss C., Spierings A.B., Schleifenbaum J.H., Wegener K. (2022). Influence of the particle size distribution of monomodal 316L powder on its flowability and processability in powder bed fusion. Prog. Addit. Manuf..

[45] Marchese G., Atzeni E., Salmi A., Biamino S. (2021). Microstructure and Residual Stress Evolution of Laser Powder Bed Fused Inconel 718 under Heat Treatments. J. Mater. Eng. Perform..

[46] Brown D.W., Anghel V., Balogh L., Clausen B., Johnson N.S., Martinez R.M., Pagan D.C., Rafailov G., Ravkov L., Strantza M. (2021). Evolution of the Microstructure of Laser Powder Bed Fusion Ti-6Al-4V During Post-Build Heat Treatment. Metall. Mater. Trans. A.

[47] Lee D., So T.Y., Yu H.Y., Kim G., Moon E., Ko S.H. (2024). Effect of Hot Isostatic Pressing and Solution Heat Treatment on the Microstructure and Mechanical Properties of Ti-6Al-4V Alloy Manufactured by Selective Laser Melting. Arch. Metall. Mater..

[48] Varmaziar S., Mostaan H., Rafiei M., Yeganeh M. (2021). Welding and Corrosion Behavior of AISI H13 Welds: The Effect of Filler Metal on the Microstructural Evolutions. Arch. Metall. Mater..

[49] Chmielewska A., Wysocki B.A., Gadalińska E., MacDonald E., Adamczyk-Cieślak B., Dean D., Świeszkowski W. (2022). Laser powder bed fusion (LPBF) of NiTi alloy using elemental powders: The influence of remelting on printability and microstructure. Rapid Prototyp. J..

[50] Wysocki B., Maj P., Sitek R., Buhagiar J., Kurzydłowski K.J., Święszkowski W. (2017). Laser and Electron Beam Additive Manufacturing Methods of Fabricating Titanium Bone Implants. Appl. Sci..

[51] Yonehara M., Ikeshoji T.T., Nagahama T., Mizoguchi T., Tano M., Yoshimi T., Kyogoku H. (2020). Parameter optimization of the high-power laser powder bed fusion process for H13 tool steel. Int. J. Adv. Manuf. Technol..

[52] Santhoshsarang D.M., Narayanaswamy S., Telasang G., Divya K., Bathe R., Samuel G.L. (2025). Additive Manufacturing of AISI H13 Tool Steel with Combinations of Higher Laser Power and Scan Speed: Microstructural and Mechanical Properties Insights. J. Mater. Eng. Perform..

[53] Tapia G., Elwany A.H., Sang H. (2016). Prediction of porosity in metal-based additive manufacturing using spatial Gaussian process models. Addit. Manuf..

[54] Gor M., Dobriyal A., Wankhede V., Sahlot P., Grzelak K., Kluczyński J., Łuszczek J. (2022). Density Prediction in Powder Bed Fusion Additive Manufacturing: Machine Learning-Based Techniques. Appl. Sci..

[55] Barrionuevo G.O., Ramos-Grez J.A., Walczak M., Betancourt C.A. (2021). Comparative evaluation of supervised machine learning algorithms in the prediction of the relative density of 316L stainless steel fabricated by selective laser melting. Int. J. Adv. Manuf. Technol..

[56] Eshkabilov S., Ara I., Azarmi F. (2022). A comprehensive investigation on application of machine learning for optimization of process parameters of laser powder bed fusion-processed 316L stainless steel. Int. J. Adv. Manuf. Technol..

[57] Mendes-Moreira J., Soares C., Jorge A.M., Sousa J.F.D. (2012). Ensemble approaches for regression: A survey. ACM Comput. Surv..

[58] Shen T., Zhang W., Li B. (2023). Machine learning-enabled predictions of as-built relative density and high-cycle fatigue life of Ti6Al4V alloy additively manufactured by laser powder bed fusion. Mater. Today Commun..

[59] Babakan A.M., Davoodi M., Shafaie M., Sarparast M., Zhang H. (2023). Predictive modeling of porosity in AlSi10Mg alloy fabricated by laser powder bed fusion: A comparative study with RSM, ANN, FL, and ANFIS. Int. J. Adv. Manuf. Technol..

[60] Amar E., Popov V., Sharma V.M., Andreev Batat S., Halperin D., Eliaz N. (2023). Response Surface Methodology (RSM) Approach for Optimizing the Processing Parameters of 316L SS in Directed Energy Deposition. Materials.

[61] Mertens R., Vrancken B., Holmstock N., Kinds Y., Kruth J., Van Humbeeck J. (2016). Influence of Powder Bed Preheating on Microstructure and Mechanical Properties of H13 Tool Steel SLM Parts. Phys. Procedia.

[62] Åsberg M., Lin F., Karlsson P., Oikonomou C., Strandh E., Uhlirsch M.K.P. (2024). A Comparative Study of the As-Built Microstructure of a Cold-Work Tool Steel Produced by Laser and Electron-Beam Powder-Bed Fusion. Metals.

[63] Moritz S., Schwanekamp T., Reuber M., Lentz J., Boes J., Weber S. (2023). Impact of in Situ Heat Treatment Effects during Laser-Based Powder Bed Fusion of 1.3343 High-Speed Steel with Preheating Temperatures up to 700 °C. Steel Res. Int..

